# Assessing Species Habitat Using Google Street View: A Case Study of Cliff-Nesting Vultures

**DOI:** 10.1371/journal.pone.0054582

**Published:** 2013-01-23

**Authors:** Pedro P. Olea, Patricia Mateo-Tomás

**Affiliations:** Instituto de Investigación en Recursos Cinegéticos, Consejo Superior de Investigaciones Científicas-University of Castilla-La Mancha-Junta de Comunidades de Castilla-La Mancha, Ciudad Real, Spain; Institut Pluridisciplinaire Hubert Curien, France

## Abstract

The assessment of a species’ habitat is a crucial issue in ecology and conservation. While the collection of habitat data has been boosted by the availability of remote sensing technologies, certain habitat types have yet to be collected through costly, on-ground surveys, limiting study over large areas. Cliffs are ecosystems that provide habitat for a rich biodiversity, especially raptors. Because of their principally vertical structure, however, cliffs are not easy to study by remote sensing technologies, posing a challenge for many researches and managers working with cliff-related biodiversity. We explore the feasibility of Google Street View, a freely available on-line tool, to remotely identify and assess the nesting habitat of two cliff-nesting vultures (the griffon vulture and the globally endangered Egyptian vulture) in northwestern Spain. Two main usefulness of Google Street View to ecologists and conservation biologists were evaluated: i) remotely identifying a species’ potential habitat and ii) extracting fine-scale habitat information. Google Street View imagery covered 49% (1,907 km) of the roads of our study area (7,000 km^2^). The potential visibility covered by on-ground surveys was significantly greater (mean: 97.4%) than that of Google Street View (48.1%). However, incorporating Google Street View to the vulture’s habitat survey would save, on average, 36% in time and 49.5% in funds with respect to the on-ground survey only. The ability of Google Street View to identify cliffs (overall accuracy = 100%) outperformed the classification maps derived from digital elevation models (DEMs) (62–95%). Nonetheless, high-performance DEM maps may be useful to compensate Google Street View coverage limitations. Through Google Street View we could examine 66% of the vultures’ nesting-cliffs existing in the study area (*n = *148): 64% from griffon vultures and 65% from Egyptian vultures. It also allowed us the extraction of fine-scale features of cliffs. This World Wide Web-based methodology may be a useful, complementary tool to remotely map and assess the potential habitat of cliff-dependent biodiversity over large geographic areas, saving survey-related costs.

## Introduction

Habitat – any part of the biosphere where a particular species can live [1]– determines the occurrence, abundance, and individual fitness of a population [2]; and hence measuring and monitoring habitat of organisms is a crucial task in ecology, management and conservation of species. Today, habitat loss and degradation are among the most serious drivers of extinction of species worldwide [3]. Consequently, the assessment of habitat across spatial scales has become a priority task for biodiversity conservation [e.g. [4].

The measurement of the quantity and quality of a species’ habitat is often a costly and time-consuming labour, becoming prohibitively expensive to collect through field-based surveys over large spatial extents [5]. Fortunately, recent advances in remotely sensed imagery and related technologies, along with the development of geographic information systems (GIS), have reduced costs and limitations associated with the collection and processing of habitat data [5], [6]. Advantages provided by remote sensing include the characterization of habitat and biodiversity over large spatial extents in a consistent manner and regularly updated [5], [7], [8]. In spite of these advances, some habitat types or habitat features have yet to be partially or completely collected on ground, with consequential associated limitations to their study over large areas.

**Figure 1:**
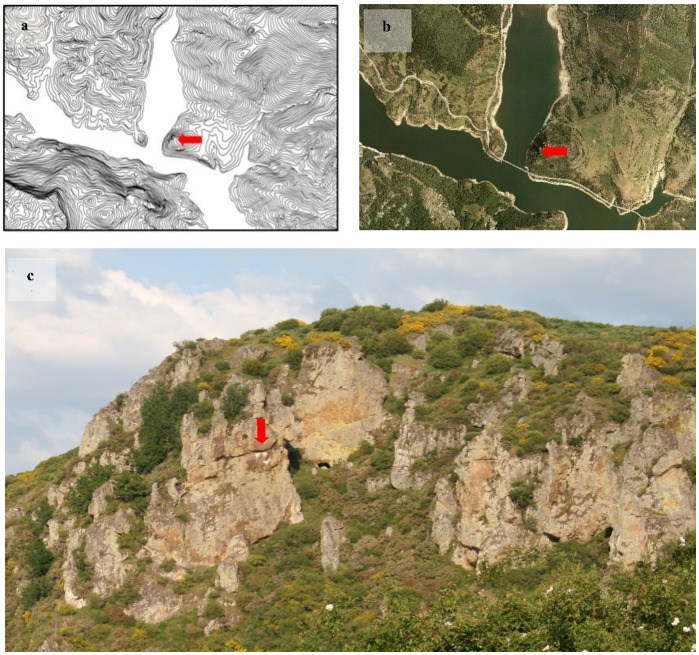
Illustrative examples of a same cliff viewed from different sources. (a) a topographical map (data source: Instituto Geográfico Nacional de España), (b) an aerial photograph (data source: Instituto Geográfico Nacional de España), and (c) a picture taken *in situ* (Autor: PMT). Red arrows indicate the location of the cliff. Similar images to b and c can also be obtained from Google Maps™ (http://goo.gl/maps/xQ4e8; Accessed: 2012-11-29), and Google Street View (Google Maps™, © Google) (http://goo.gl/maps/hz2LX; Accessed: 2012-11-29).

Cliffs are steep faces that create abrupt discontinuities in the landscape, shaping inaccessible habitats and least-disturbed ecosystems, which support a rich biodiversity (from ancient communities of plants to threatened raptors; [9], [10], [11]). For example, 20 (44%) of the 45 diurnal species of birds of prey in Europe use cliffs for nesting obligatorily (17.7%) or facultatively (26.7%) (authors’ unpublished data; [12]). Because of their principally vertical structure, cliffs have not been easy to identify and assess by remote sensing technologies, which are based on a bird-eye perspective (Figure 1, see [6] for an approach to estimate cliff availability). This drawback has posed a challenge to adequately deal with cliff habitat for many researches and managers working with cliff-dwelling species [9], [10], [11]. Technologies recently launched could however assist in remotely collecting cliff habitat information, reducing survey-related costs and limitations. Finding the most cost-effective methods for biodiversity monitoring and conservation is necessary, as funds available for these activities are limited [13].

Google Street View is a freely available tool incorporated in Google Maps and Google Earth® that provides panoramic views along many streets and roads around the world (http://en.wikipedia.org/wiki/Google_Street_View). It was launched in May 2007 in the United States and in July 2008 in Europe, and since then has expanded to cover a wide net of cities and rural areas worldwide. This application allows users free viewing of georeferenced, high-resolution full-color images in a continuous way along most of the roads from a pedestrian level. Accordingly, it may be a useful tool to remotely identify and evaluate some habitats, such as cliffs, at a finer scale than that shown so far (Figure 1). Despite its potential for the evaluation of diverse environments, as far as we know, Google Street View has yet been underused in research. Most works so far using Google Street View have been developed in the categories of health sciences and in social sciences and humanities, but no study has been conducted in life sciences (as assessed from a search on Scopus from 1960 to 21^st^ February 2012 for “google street view” or “street view” in the fields of “abstracts, titles and keywords”).

In this paper we explore the feasibility of Google Street View as a useful tool to identify and assess the nesting habitat of two cliff-nesting species, namely the griffon vulture *Gyps fulvus* and the globally endangered Egyptian vulture *Neophron pernocpterus*. We evaluated two main potential uses of Google Street View to ecologists and conservation biologists: i) remotely identifying a species’ potential habitat to assist in the subsequent sampling design and ii) extracting fine-scale information from habitat data for potential use in habitat selection studies (or species’ distribution models, SDMs). Both tasks account for much of the activity developed by wildlife researchers and managers.

## Methods

### Study Area

**Figure 2:**
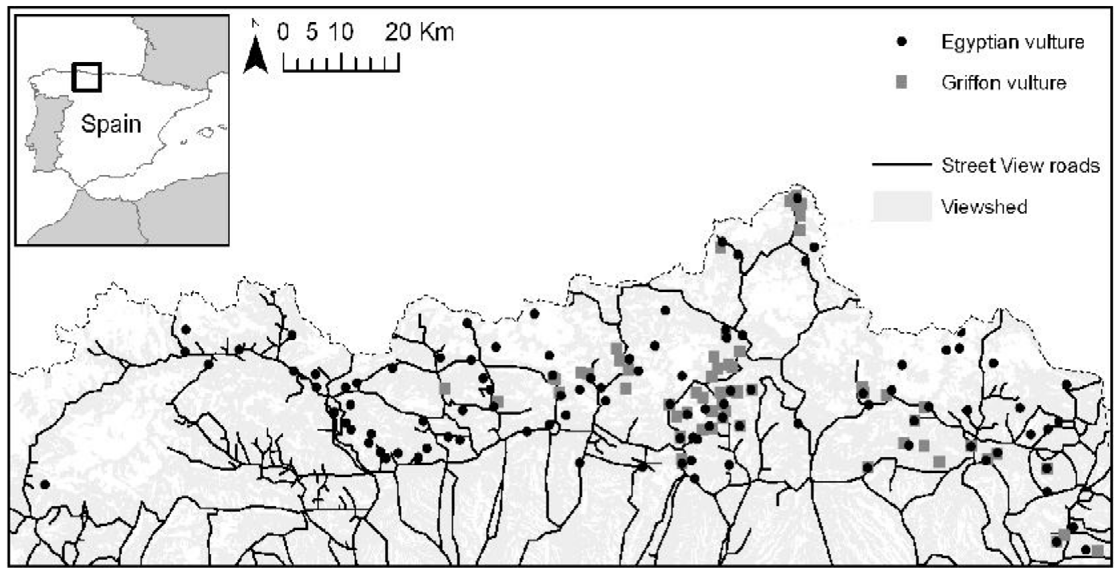
Location of the study area in north-western Spain. Sixty five percent of the study area was potentially visible (bright grey) from the paved roads covered by Google Street View (black lines). The location of the cliffs used by griffon and Egyptian vultures for nesting is also shown. The dotted line indicates the northern limit of the study area (in the black square in the inset).

The study area covers 7,000 km^2^ on the south slope of the Cantabrian Mountains, in north-western Spain (León and Palencia provinces; Figure 2). This area is covered by 3,905 km of paved roads and has a complex topography, with elevations ranging between 340–2,648 m above sea level. Rocky cliffs, mainly of limestone, are abundant all over the study area [14].

### Study Species

The two study species are obligated cliff-nesters. The Egyptian vulture is a medium-sized territorial scavenger distributed from the Mediterranean countries to India and South Africa. This species is classified as *Endangered* by the IUCN [15]. Spain holds the most important population in Europe, comprising up to 44.5% of the breeding population [15], [16]. In Spain, the species occupy very different habitats, from plains to middle and high mountains [17]. The breeding pairs arrive from their winter grounds in Africa in early March and remain in the territories until mid-September. Nesting cliffs are generally used year after year [18], [19]. The nests are usually built in caves, and more rarely on ledges or crevices. In the study area, Egyptian vultures prefer to nest in caves with vegetation at the entrance [19].

The griffon vulture is a colonial cliff-nesting scavenging raptor widely distributed from the Mediterranean countries to India, also occupying also areas in the north of Africa [20]. The species is classified as of *Least Concern* by the IUCN [20], but it is locally threatened in some regions where recovery programmes are carried out [21]. The species use caves, ledges and crevices to install their nests. Nests can be close to each other (i.e. a few meters). Griffon vultures breed mainly in colonies that range from a few to hundreds of pairs [17]. In our study area, colony size ranged from 2 to 20 breeding pairs (mean ± SE = 6±1); solitary nests (*n* = 2) were also taken into account.

### Procedure

**Figure 3:**
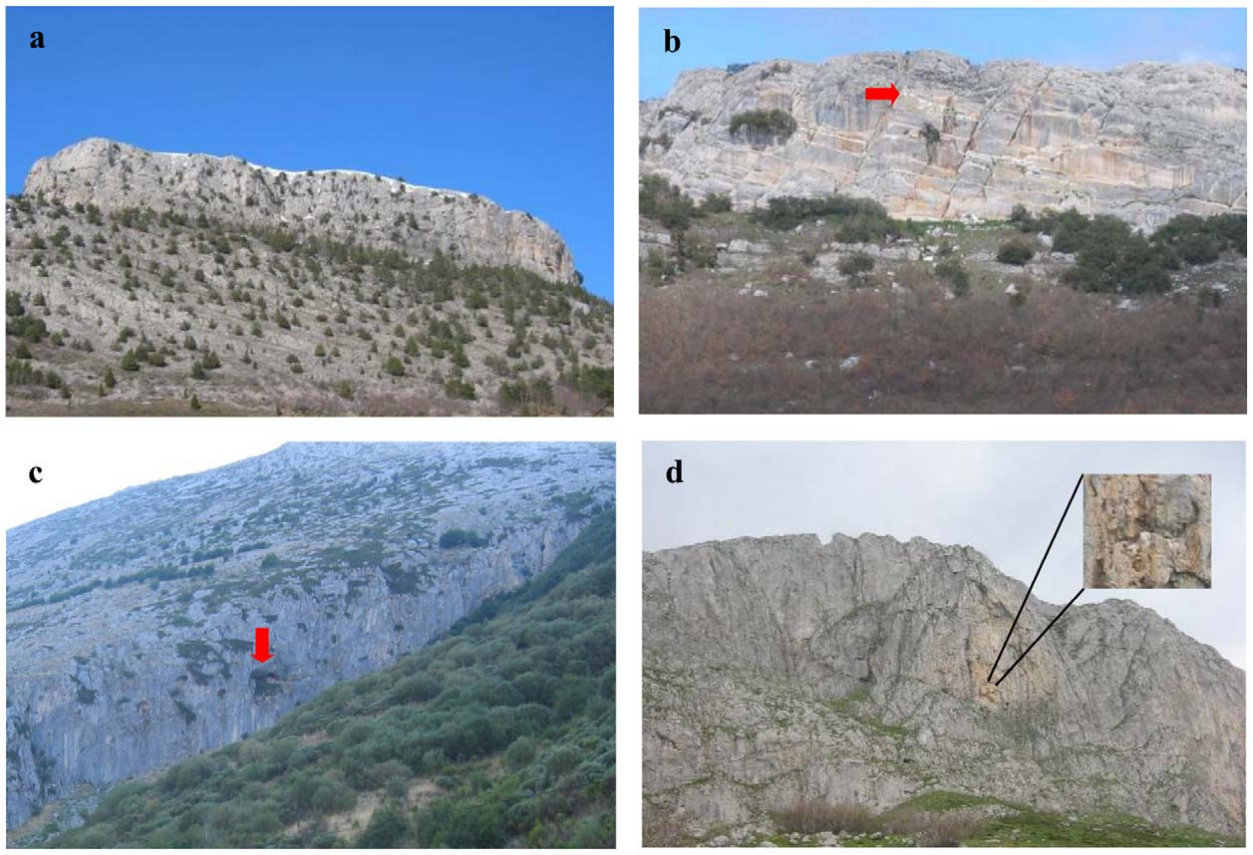
Nesting cliffs used by the griffon and the Egyptian vultures in the study area. Caves and white drops are highlighted with red arrows or expanded by zooming. All the four images are photographs taken by authors *in situ*. These same cliffs can be remotely observed by using Google Street View (Google Maps™, © Google) [Figure 3a: http://goo.gl/maps/zmBya; Figure 3b: http://goo.gl/maps/F1Byx; Figure 3c: http://goo.gl/maps/b3ROu; Figure 3d: http://goo.gl/maps/bKNZT; All the images accessed: 2012-11-29].

In Google Maps or Google Earth an orange “pegman” icon appears (Figure 3). By dragging it onto a location on the map, you can view and navigate within road-level imagery using the Street View feature (see http://maps.google.es/support/bin/answer.py?answer=144358 for more details). We conducted visual inspection of the Google Street View imagery searching for cliffs. Dates of the imagery provided by Google Street View were between August and October 2009.

#### Remote identification of potential habitat

To assess the usefulness of Google Street View to assist in the initial design of species censuses (usefulness (*i*), see Introduction), we first randomly selected seven 10×10-km UTM squares entirely located within the study area. Four observers inspected each of these seven squares looking for rocky cliffs using Google Street View, and noted the time spent on this task for each square. The four observers were: one expert on vulture census and knowledgeable of the study area; one expert on cliff raptors but not familiar with the study area; and two non-experts in censusing vultures also unfamiliar with the study area. We calculated the area potentially surveyed from roads covered by Google Street View within each 10×10-km UTM square by using the Viewshed utility of ArcGIS 10 (Environmental Systems Research Institute Inc., Redlands, California, US). The distribution of paved roads covered by Google Street View was obtained at http://maps.google.es/intl/es/help/maps/streetview/learn/where-is-street-view.html and implemented in a GIS.

At the same time, we estimated the virtual time spent looking for cliff habitat in the same seven squares studied with Google Street View, as if the field survey was entirely performed by car. On-ground survey by car would cover all the paved and unpaved roads in each square at an estimated mean speed of 30 km h^-1^. We calculated the final area surveyed by using the Viewshed utility described above. The distribution of paved and unpaved roads was obtained from official databases (©Instituto Geográfico Nacional de España) and aerial photographs, and implemented in a GIS. Monetary costs of the virtual on-ground survey were estimated by assuming a mean consumption of 0.19 euro km^−1^ (Real Decreto 462/2002) [22]. We compared the time spent between observers looking for cliffs in the squares using Google Street View by applying pair-wise comparisons of Wilcoxon signed rank paired tests; we used the same test for examining differences of time spent looking for cliffs using Google Street View and virtual on-ground surveys.

#### Digital Elevation Model (DEM) vs Google Street View

Cliffs can be located through the conventional analysis of the slope of the terrain in GIS (Figure 1) [14]. We aimed to compare the ability of DEM to identify cliffs with that of Google Street View. We used a high resolution (i.e. 5 m pixel) DEM to obtain the slope values for the study area in ArcGIS 10. To select the threshold values above which classify a location as a cliff, we considered the slope of all the vulture breeding cliffs recorded in the study area (i.e. our habitat of interest; *n* = 148 cliffs) [14]. On this distribution of slopes, we selected three different thresholds [14]: the minimum slope value (0.34), 25^th^ (0.63) and 50^th^ percentile (0.68). These threshold values were used to obtain maps (i.e. Smin, S25^th^ and S50^th^, respectively) of potential cliffs.

To assess accuracy in the identification of cliffs, we randomly assigned a total of 100 points (i.e. field test samples, 50 on cliffs and 50 on non-cliff habitat) at the seven 10×10-km UTM squares previously selected (see above; 14–15 points per square). These points were located within the area potentially surveyed with Google Street View (see above). This allowed a better comparison between methods (i.e. DEM maps and Google Street View). Ground truthing for points was determined through field surveys. Overall accuracy, producer and user accuracy, omission and commission error rates, and Cohen’s Kappa coefficients were calculated for each method, i.e. DEM maps (Smin, S25th and S50th) and Google Street View. Overall accuracy is the division of the total number of correctly classified points by the total number of points; producer’s accuracy is the percentage of field points of a category which are correctly classified as that category by the method used (or map derived); user’s accuracy is the percentage of points of a category derived from the method (or map) which are really in that category [23]. Omission errors are false negative predictions, while commission errors are false positive predictions. The Cohen’s Kappa coefficient indicates the degree of agreement between the classification results and the on-ground (reference) points. Cohen’s Kappa coefficients were performed with the *irr* package [24] in R [25].

#### Obtaining fine-scale habitat characteristics

In order to assess the usefulness of Google Street View to obtain fine-scale habitat characteristics (usefulness (*ii*), see Introduction), we first determined the percentage of nesting cliffs known to be used by griffon and Egyptian vultures that we were able to unequivocally identify through Google Street View. Nesting cliffs known to be used by griffon and Egyptian vultures were available from previous studies [14], [16], [19], [20], [26], [27], [28], [29], [30], [31], [32], [33], [34]. For a detailed description of the census methodology of both species, see, for example, [34] for the Egyptian vulture and [14] for the griffon vulture. For each of these known vulture nesting cliffs, one observer experienced in censusing vultures and knowledgeable of the study area (see above) took the coordinates of the occupied cliffs, searched for these cliffs using Google Street View and assessed whether or not he/she was able to unequivocally identify the cliffs using Google Street View (i.e. clearly see at least 80% of the cliff previously identified through field surveys; see Figure 3 for examples). If the cliffs were identified through Google Street View by visual inspection, the observer noted whether or not he/she could also see caves, vegetation (i.e. shrubs and/or trees on the cliff) and white spots of excrements. These characteristics, which can indicate a higher probability of occupancy of those cliffs by the study species [19], are used here as a way to assess its usefulness to extract fine-scale habitat information (Figure 3). The observer also noted the type of substrate (i.e. limestone or non-limestone) of the nesting cliffs visually identified from Google Street View. Distances from nesting cliffs to the nearest road covered by Street View were calculated in ArcGIS 10.

## Results

Of the 3,905 km of paved roads existing in the study area, 49% (i.e. 1,907 km) were covered by Google Street View. The potential visibility (calculated from the Viewshed utility in ArcGIS 10; see above) covered by Google Street View was 65% (4,550 km^2^) of the whole surface of the study area (Figure 2). This potential visibility ranged between 20.6 and 76.4% per 10×10-km square with a mean of 48.1±7.6% (SE) (Table 1). As the virtual on-ground survey by car included dirt roads, the potential visibility covered by car was significantly greater (mean: 97.4±0.98% per 10×10-km square, range: 93.4–99.9%) than that of Google Street View (paired t-test, *t* = −6.30, *p = *0.0007). Time spent looking for cliffs using Google Street View was not significantly different between observers (pair-wise comparisons; Wilcoxon signed rank paired test, *V = *12–18, *p = *0.21–0.94). Time spent looking for cliffs was significantly lower using Google Street View (total mean of the four observers: 0.91±0.08 min km^−2^ of surveyed area, range: 0.24–1.70 min km^−2^) than using on-ground surveys by car (mean: 3.97±1.1 min km^−2^, range: 1.82–10.48 min km^−2^; Wilcoxon signed rank paired test, *V = *0, *p = *0.016). The cost of looking for cliffs on-ground was of 0.38±0.11 euro km^−2^ of surveyed area (range: 0.17–1.00 euro km^−2^). The surveyed area using Google Street View encompassed 49.5±7.8% (range: 21–76%) of that covered by on-ground survey. Thus, if this area coincident between both methods could be covered by Google Street View instead of by on-ground survey by car, it would save 175.1±96.1 min and 20.7±9.4 euro per 10×10-km square; that is, 12,262±6726 min (204.4 hours) and 1,447±657 euro for the whole study area, saving 36.1±7.9% in time and 49.5±7.8% in costs with respect to the on-ground surveys by car only.

**Table 1:** Mean time and monetary cost per km^−2^ of surveyed area (viewshed) looking for suitable habitat for cliff-nesting vultures by using different methods.

	On-ground mean ± SE (range)	Google Street View mean ± SE (range)	Combined mean ± SE (range)
Viewshed (%)	97.4±0.98 (93.4–99.9)	48.1±7.6 (20.6–76.4)	97.4±0.98 (93.4–99.9)
Time (min km^−2^)	3.97±1.11 (1.82–10.48)	0.91±0.08 (0.24–1.70)	2.15±0.30 (0.77–3.12)
Cost (Euro km^−2^)	0.38±0.11 (0.17–1.00)	0.00	0.17±0.07 (0.06–0.29)

Values were calculated from a sample of seven 10×10-km squares randomly located within the study area. The percentage of square surface prospected with each method is also shown (Viewshed). Time costs for Google Street View are mean values obtained from four different observers (see text for further explanations).

Google Street View had an overall accuracy in classifying cliffs of 100% (Cohen’s Kappa = 1) (Table 2). For the DEM maps, Smin correctly classified the 94% (Cohen’s Kappa = 0.89) of the ground points, S25^th^ correctly classified 79% (Cohen’s Kappa = 0.65), and overall accuracy for S50^th^ was of 72% (Cohen’s Kappa = 0.56) (Table 2).

**Table 2:** Results of the accuracy assessment of different methods.

Ground points	Results of classification	SV	Smin	S25th	S50th
Cliffs (*n* = 50)	Correct (Incorrect)	50 (0)	49 (1)	29 (21)	22 (28)
No-cliff (*n* = 50)	Correct (Incorrect)	50 (0)	45 (5)	50 (0)	50 (0)
	Overall accuracy (%)	100	94	79	72
	Producer’s accuracy (%)	100	98	58	44
	User’s accuracy (%)	100	90	100	100
	Omission error rate	0	0.02	0.42	0.56
	Commission error rate	0	0.10	0	0
	Cohen’s Kappa coefficient	1	0.89	0.65	0.56

One hundred points were randomly chosen within the study area (50 cliffs and 50 non-cliff, i.e. ground truthing) against which the results of the classification of each method were compared: Google Street View (SV) and three DEM-based maps with different thresholds of slope (Smin, S25th and S50th, see text for more details). The table shows also overall accuracy, producer and user accuracy, omission and commission error rates and Cohen’s Kappa coefficients for each method.

In the study area there are 148 nesting cliffs known to be occupied by vultures: 58 (39%) by griffon and 104 (70%) by Egyptian vulture; 14 (9%) cliffs were shared by both species. From these 148 nesting cliffs, we observed 97 (66%) cliffs through the Google Street View imagery: 37 (64%) out of 58 nesting cliffs of griffon vulture and 68 (65%) out of 104 of Egyptian vulture (the between-species difference in the number of detected cliffs was not significant, 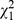 = 0.01, *P* = 0.93) (Table 3).

**Table 3:** Number of cliffs used for breeding by griffon and Egyptian vultures which were identified with Google Street View.

	No. cliffs	Identified in GoogleStreet View
Total	148	97 (66%)
Griffon vulture	58	37 (64%)
Egyptian vulture	104	68 (65%)
Both species	14	8 (57%)
Cliffs with white spots	114	46 (40%)
Cliffs with caves	88	25 (28%)
Cliffs with vegetation	123	80 (65%)

The nesting cliffs observed through Google Street View laid to a significantly shorter mean distance to the nearest road covered by Google Street View (mean ± SE: 955±67 m, range: 43–3,729 m, *n = *97) than that of the unobserved cliffs (2,170±210 m, range: 310–8,782 m, *n = *51; *t = *5.51, *P*<0.001). The nesting cliffs of griffon vulture identified with Google Street View were observed at a larger average distance from the nearest road covered by Google Street View (1,076±93 m, *n = *29) than those of the Egyptian vulture (839±86 m, *n = *60; although non-significant: *t = *1.87, *P = *0.065; same cliffs used by both species were excluded from the analysis). This between-species difference in cliff identification was not due to the cliffs used by griffon vulture being farther from roads covered by Google Street View than that of Egyptian vulture (mean for griffon vulture cliffs: 1,432±141 m, *n = *44 *vs* Egyptian vulture: 1,336±140 m, *n = *90; *t = *0.48, *P = *0.63; same cliffs used by both species were excluded from the analysis).

We determined correctly the type of substrate in 100% (*n = *97) of the nesting cliffs detected via Google Street View, white spots of excrement were observed in 48% (*n = *46), caves in 26% (*n = *25) and vegetation in 65% (*n = *80). Field surveys showed that white spots were observed in 77% (*n = *114), caves in 59% (*n = *88) and vegetation in 76% (*n = *123) of the nesting cliffs. Therefore, using Google Street View we detected white spots, caves and vegetation in 40%, 28% and 65% respectively, of the subset of cliffs with caves, white spots and vegetation registered in the field surveys (Table 3).

## Discussion

Ecosystem study and management require the collection of spatially-explicit detailed information for mapping and assessing habitats and biodiversity over large areas, but this information is usually difficult and costly to gather through field-based techniques [35], [36]. Remote sensing through airborne or satellite sensors has greatly contributed to addressing this need [35]. Yet, certain attributes of the landscape and fine-scale habitat elements are undetectable by remote sensing, thereby being still largely dependent on field-based data for their characterization and thus greatly limiting the spatial extent to study. Cliffs are understudied, species-rich ecosystems [9], [10], whose identification and assessment in a landscape through remote sensing or DEM maps is not straightforward, and thus characterization of this ecosystem for studying cliff biota has had to be generally conducted by costly on-ground surveys (e.g. [9], [10], [11], [14], [16], [19], [30], [31], [32], [33], [34]). In this paper we show that a considerable portion (65%) of the area prospected to locate suitable habitat for two cliff-nesting vulture species could be remotely surveyed and that an important percentage of their nesting cliffs could be observed (66%) and evaluated for features (28–100%) by a surveyor using Google Street View. Furthermore, although the conventional method which used digital elevation models (DEMs) provided good results regarding cliff identification (up to 95% of correctly classified cliffs), Google Street View outperformed the DEMs in accuracy (Table 2). All of this suggests that Google Street View may be a useful tool to assist in habitat surveys and census of cliff-related biodiversity, reducing also survey-related costs (e.g. transportation time and mileage, fossil fuel consumption [37]). Reducing the costs associated with (habitat) data collection is essential in the worldwide context of limited resources for biodiversity research and conservation [13]. The use of this web-based tool can be quite useful on a landscape scale. It would enable the design of more efficient fieldwork on any cliff-dependent species at the early stages of the study by focusing and prioritizing on more suitable areas and/or cliffs or in remote areas away from paved roads, while avoiding less suitable ones (e.g. areas without cliffs), thus saving both time and money. In our study, Google Street View only allowed covering between 21 and 76% of the area of each 10×10-km square, so it obligated combining the use of this web-based tool with other method(s) to completely survey the square. Our results suggest that the use of Google Street View in conjunction with high-performance DEMs (e.g. Smin) could be highly useful as a first coarse-scale approach to identify and map cliffs over large geographic areas. Nonetheless, on-ground data (e.g. surveys by car) should be collected in the area uncovered by Google Street View to refine the cliff map, as DEM misclassify a variable percentage of locations (Table 2). The incorporation of Google Street View to this study would save 36% in time and 49.5% in monetary costs with respect to the car on-ground survey only. Note that we did not take into account costs of travel from the point of origin to the squares, so the costs saved by using Google Street View would be greater. Although these particular figures are site-specific, they illustrate the usefulness of this web-based tool in planning field surveys.

Once the nesting sites are known –which can only be reliably attained by on-ground surveys in our study species (e.g. [34]) – Google Street View can still assist researchers and managers who can also remotely obtain fine-scale features of used and available cliffs to inform studies of habitat selection. This is an important added advantage of Google Street View that is not currently provided by other remote-sensing techniques. Nowadays, much existing information consists of species’ occurrence data with georeferencing records in digital databases (e.g. Global Biodiversity Information Facility: http://www.gbif.org/), making it widely available to be used in habitat selection models or SDMs [38], [39] for which Google Street View may aid to remotely extract free-cost, fine-scale habitat information from these occurrence sites (Figure 3). Our study adds to the small but increasing body of evidence proving the usefulness and potential of the World Wide Web-based tools for surveys on species ecology and conservation (e.g. [40], [41], [42], [43]). Google Street View offers an inexpensive, rapid means for obtaining fine-scale environmental information for large geographic areas, and allows similar advantages to those provided by others remote sensing techniques based on airborne and satellite sensors ([7], [8]).

Nonetheless, neither all the study area could be surveyed (65%) nor all the nesting cliffs known to be occupied by vultures could be identified via Google Street View (i.e. 66%). This spatially uneven coverage establishes a difference between Google Street View and other remote-sensing techniques, which sample the terrain in a spatially complete manner ([7], [8]). Moreover, only a fraction of the nesting-cliffs could be evaluated for some fine-scale characteristics (e.g. presence of caves, 28%; bird depositions, 40%; vegetation, 65%). Therefore, Google Street View is not currently a substitute for cliff habitat on-ground studies, but rather a useful complement to them (see above). It is expected, however, that the usefulness of this tool will increase in the future if the coverage presently available on Google Street View increases (e.g. only the 48.8% of the paved roads in our study area is currently covered), and especially if it extends to dirt roads (e.g. using trikes; http://maps.google.com/intl/en/help/maps/streetview/technology/cars-trikes.html). This expansion into dirt roads would solve one of the limitations we have found in this work: i.e. the impossibility of assessing those cliffs located far away from the paved roads. In fact, our results indicate that the distance to which the cliffs are located from the roads covered by Street View was a limiting factor to study cliffs with this technology, as these distances were shorter for identified than for unidentified cliffs. In our study area, this distance limit to which cliffs become unidentifiable could be around 1 km from the road covered by Street View, as suggested by our results (i.e. most of the identified cliffs lay within around that distance; median: 800 m; 75^th^ percentile = 1,173 m). Although not addressed in this paper, multiple factors could affect variation in the distance within which the cliffs can be identified with Street View (e.g. vegetation structure), but obviously the size of the cliff to identify has to be important. This idea is supported by our results showing that the species that use larger nesting cliffs (i.e. the griffon vulture; authors’s unpublished data) [14], [21] registered a greater mean distance from the road to the identified cliff. Other limitations of this method were those related with meteorological and light conditions (e.g. fog, cloudy, backlighting) under which Street View imagery were taken, which prevented us from adequately evaluating the 4.1% of the cliffs. In addition, Google imposes restrictions on the use of Street View images (http://support.google.com/maps/bin/static.py?hl=en&ts=1342531&page=ts.cs ). These images may only be shared in publications via direct links (see Fig. 1 and 3) or through an application programming interface (API) (https://google-developers.appspot.com/maps/). Therefore, Google Street View images that are shared via direct links in published studies may not be permanently accessible (e.g. they may be periodically updated by Google or subject to change in the access site).

We have tried to keep the assessment of cliff features simple, but other cliff features can also be assessed or tried (e.g. size of the cliff, number of caves, ledges and crevices). In fact, we think that measures of height and width as well as surface of the cliffs or parts of them (e.g. size of the caves) could be obtained, as evidenced by the recent development of techniques for measuring objects such as building facades from Street View imagery [44], [45]. Once implemented, this new technique may provide a valuable tool to the standard assessment of cliff size, as it is currently a very difficult and inaccurate measure to obtain on ground. Its application would increase the quality of the information on cliff habitat improving the studies on selection of habitat for cliff-dependent species.

Cliffs are expected to change little over time and so they are a type of habitat adequate to study with online tools such as Google Street View, which are not as rapidly updated as other remote sensing technologies (e.g. airborne and satellite imagery) [5]. This web tool has the potential to be also useful in detecting other biodiversity elements of cliff ecosystems such as plants or ancient trees, [9], [10], [11] as well as other types and features of habitat valuable for other species (e.g. vertical structure and composition of the vegetation along the roads, detection of nesting sites occupied by conspicuous species breeding in cities and close to roads such as the rook *Corvus frugilegus;* authors, pers. obs.). It could also have potential to be applied in other fields such as risk assessment of rock falls from natural rock slopes [46], or in environmentally friendly cliff road construction [47].
